# Safety Analysis Using Lebesgue Strain Measure of Thick-Walled Cylinder for Functionally Graded Material under Internal and External Pressure

**DOI:** 10.1155/2013/676190

**Published:** 2013-08-05

**Authors:** A. K. Aggarwal, Richa Sharma, Sanjeev Sharma

**Affiliations:** Department of Mathematics, Jaypee Institute of Information Technology, A-10, Sector 62, Noida 201307, India

## Abstract

Safety analysis has been done for thick-walled circular cylinder under internal and external pressure using transition theory which is based on the concept of generalized principal Lebesgue strain measure. Results have been analyzed theoretically and discussed numerically. From the analysis, it can be concluded that circular cylinder made of functionally graded material is on the safer side of the design as compared to homogeneous cylinder with internal and external pressure, which leads to the idea of “stress saving” that minimizes the possibility of fracture of cylinder.

## 1. Introduction

The constantly increasing industrial demand for axisymmetrical cylindrical and spherical components or elements of them has concentrated the attention of designers and scientists on this particular area of activity. The research on the prediction of stresses in thick-walled hollow circular cylinder has never ceased because of the importance of these basic structures in numerous mechanical, civil, electrical, and computer engineering applications. These days in nuclear industry, cylinders subjected to internal and external pressure have become a point of interest due to their application to advanced small and medium-sized light water reactors. For example, steam generator tubes, in which primary coolant flows outside the tubes while secondary water flows inside the tubes are typical examples of cylinders under internal and external pressure. Another example is pipelines under seawater to transport gas, oil, and so forth. Now for design and integrity evaluation of a cylinder under internal and external pressure, one should carefully consider the failure characteristics of a cylinder under internal and external pressure. The failure mechanisms of such type of cylinder might be quite different from those of a typical one under internal pressure. Upon the estimation of load carrying capacity of these thick-walled cylinders under internal and external pressure and combined loading, many numerical and experimental works have been also made to propose relevant design criteria of thick-walled cylinders subjected to internal and external pressure. Plane strain and plane stress analytical solutions of thick hollow cylinder problems in the elastic stress state have been available for many years in standard and advanced textbooks [1–4]. Thick-walled circular cylinder subjected to internal and external pressure is widely used in various industries. In general vessels under high pressure require a strict analysis for an optimum design for reliable and secure operational performance and thus efforts were continually made to increase reliability. Solutions have been obtained either in analytical form or with numerical implementations. The literature includes solutions of Chen [5] who suggested an finite difference approach for the axisymmetric plane strain problem beyond the elastic limit while Durban and Kubi [6] suggested an analytical method for pressurized elastic-plastic tubes in plane strain. Jahed and Dubey [7] proposed a numerical method for solution for elastic-plastic tubes using total deformation theory of plasticity. Parker [8] implemented a numerical procedure to calculate pressure and associated residual stress fields for open cylinder. Dubey et al. [9] obtained solutions for an elastic-plastic work hardening model using piecewise linearization of constitutive law. Olszak and Urbanowski [10] calculate the stresses for nonhomogeneous thick-walled elastic-plastic cylinder subjected to internal pressure while Hodge and Balaban [11] calculated the stresses for rotating cylinder. Sharma [12] analyzed thick-walled cylinders under internal pressure for isotropic nonhomogeneous elastic-plastic states using transition theory. This paper is an extension of Sharma [12] to include the effect of external pressure for functionally graded material because these days cylinders made of functionally graded material under internal and external pressure are an important design consideration in nuclear industry.

## 2. Generalized Lebesgue Strain Measure

The classical theory of elasticity and plasticity divides the spectrum of deformation of solids into two different states, one of which is elastic and other one is plastic. In classical theory, both field equations are solved separately and later joined together by yield condition. As in the behavior of materials, perfect elasticity and perfect plasticity are extremes, but no one can draw a sharp line between these two states. It is therefore natural to expect that there should be a transition state, and at this transition, a continuum approach necessarily means the introduction of nonlinear measure. But in classical mechanics, the ordinary measure has been found sufficient and so no extension has been made. This is because of the reason that, in classical mechanics, field equation for elastic and plastic regions is calculated separately and then connected by yield criterions which is an assumption. Also, if in a very small interval, the number of fluctuation is very large, the ordinary measure based on Riemann integral fails and measures like that of Lebesgue have been used. This generalized Lebesgue measure gives very satisfactory results in the problems like that of plasticity and creep. The generalized Lebesgue strain measure helps to bridge the gap between microscopic and macroscopic descriptions of physical system and eliminate semiempirical conditions like that of Tresca's and von-mises, creep strain laws, that is, Nortan's law which provides a coordination between the theoretical and experimental results. Seth [13] has defined the generalized principal strain measure *ε*_*ii*_ by taking the Lebesgue integral of the weighted function *ε*_*ii*_ = ∫_0_^*e*_*ii*_^*A*^^[(1−2_*e*_*ii*__^*A*^)]^(*n*/2)−1^ *de*_*ii*_^*A*^ = (1/*n*)[1 − (1−2*e*_*ii*_^*A*^)^(*n*/2)−1^], where *n* is the measure and *e*_*ii*_^*A*^ is the principal Almansi strain component. 

## 3. Objective

In this paper, our aim is to calculate safety factor for thick-walled cylinder made of functionally graded material under internal and external pressure using Seth's transition theory [12, 14–16]. The stresses are calculated for transition as well as for fully plastic state. The constitutive equations for both transition and fully plastic states are also derived from the results. Non-homogeneity is taken as the compressibility of material in the cylinder as

(1) $$\begin{array}{c} C=C_{0}r^{-k}, \end{array}$$

where *a* ≤ *r* ≤ *b*; *C*_0_ and *k*(≤0) are constants.

Results obtained have been discussed numerically and depicted graphically. 

## 4. Mathematical Formulation

Consider a nonhomogeneous thick-walled circular cylinder of internal and external radii *a* and *b*, respectively, subjected to internal pressure *p*_1_ and external pressure *p*_2_. The non-homogeneity in the cylinder is due to variable compressibility *C* = *C*_0_ *r* ^−*k*^. The cylinder is taken so large that plane transverse sections remain plane during the expansion, and hence the longitudinal strain is the same for all elements at each stage of the expansion. In cylindrical polar coordinates the displacements are given by [14–16]

(2) $$\begin{array}{c} u=r\left(1-\beta \right);\;\;v=0,\;\;w=dz, \end{array}$$

where *β* is a function of $r=\sqrt{x^{2}+y^{2}}$ and *d* is a constant.

The generalized strains are

(3) err=1n[1−(rβ′+β)n];  eθθ=1n[1−βn];ezz=1n[1−(1−d)n];  erθ=eθz=ezr=0,

where *n* is the measure and *β*′ = *dβ*/*dr*. 

For isotropic material, the stress-strain relation in elastic region is given by

(4) $$\begin{array}{c} T_{ij}=\lambda \delta_{ij}I_{1}+2\mu e_{ij};\;\left(i,j=1,2,3\right), \end{array}$$

where *I*_1_ = *e*_*kk*_; *T*_*ij*_,  *e*_*ij*_ are stress and strain tensors, respectively, and *δ*_*ij*_ is Kronecker's delta.

Equations of equilibrium are all satisfied except

(5) $$\begin{array}{c} \frac{d}{dr}\left(T_{rr}\right)+\frac{\left(T_{rr}-T_{\theta \theta }\right)}{r}=0. \end{array}$$

A nonlinear differential equation in *β* has been obtained by substituting (4) in (5), as

(6) nPβ(P+1)n−1dPdβ =[r(μ′μ−C′C)[{(3−2C)−(1−C)(1−d)n}1βn−(1−C)−(P+1)n]+C×[1−(P+1)n]+rC′[1−{2−(1−d)n}1βn]−nP[(1−C)+(P+1)n]],

where *rβ*′ = *βP*; *C* = 2*μ*/(*λ* + 2*μ*).

The transition point of *β* in the previous equation is *P* → −1 and *P* → ±*∞*.

The boundary conditions are given by

(7) Trr=−p1 at  r=a;Trr=−p2 at  r=b.

In the cylinder, resultant axial force is given by

(8) $$\begin{array}{c} L=2\pi \int_{a}^{b}rT_{zz}dr. \end{array}$$

## 5. Method of Solution

As elastic state can go to plastic state under external loading through a transition state and we consider only the principal stresses. Therefore the transition can take place either through the principal stresses *T*_*rr*_ or *T*_*θθ*_ becoming critical or through the principal stress difference *T*_*rr*_ − *T*_*θθ*_ becoming critical. It has been shown that the asymptotic solution through the principal stress leads from elastic state to plastic state at transition point *P* → ±*∞*. For finding the plastic stress at the transition point *P* → ±*∞*, we define the transition function *R* [14–16] in terms of *T*_*rr*_ as

(9) $$\begin{array}{c} R=T_{rr}-\frac{\lambda }{n}k\equiv \frac{2\mu }{Cn}\left[C-\beta^{n}\left\{\left(1-C\right)+{\left(P+1\right)}^{n}\right\}\right]. \end{array}$$

Taking the logarithmic differentiation of (9) with respect to *r*, we get

(10) ddrlog⁡R=[rC′(1+βn)−nPβn+1(P+1)n−1dPdβ−nPβn  ×{(1−C)+(P+1)n}+r(μ′μ−C′C)  ×{C−βn[(1−C)+(P+1)n]}]  ×(r[C−βn{(1−C)+(P+1)n}])−1.

Take the asymptotic value *P* → ±*∞* of (10) after substituting  *dP*/*dβ* from (6). This on integration gives

(11) $$\begin{array}{c} R_{1}=A\exp f\left(r\right), \end{array}$$

where *A* is a constant of integration and *f*(*r*) = −∫(*C*/*r*)*dr*.

By using (7) in (9) and (11), we get

(12) $$\begin{array}{c} T_{rr}=A\left[\exp f\left(r\right)-\exp f\left(b\right)\right]-p_{2}. \end{array}$$

Using (12) in (5), we get

(13) $$\begin{array}{c} T_{\theta \theta }=A\left[\left(1-C\right)\exp f\left(r\right)-\exp f\left(b\right)\right]-p_{2}. \end{array}$$

Equations (4) yields

(14) Tzz =(1−C2−C)(Trr+Tθθ)+Cλ(1−C)(3−2C2−C)   ×(p2−p1)/2π−∫ab(rc(1−c)/(2−c))(Trr+Tθθ)drλ∫ab(rc(3−2c)/(1−c)(2−c))dr.

Taking the non-homogeneity in the cylinder due to variable compressibility

(15) Trr=A1[exp⁡(c0r−kk)−exp⁡(c0b−kk)]−p2;Tθθ=A1[(1−c0r−k)exp⁡(c0r−kk)−exp⁡(c0b−kk)]−p2;Tzz=(1−c0r−k2−c0r−k)(Trr+Tθθ)+λc0r−k(1−c0r−k)(3−2c0r−k2−c0r−k)ezz.

(16) Also,  Tθθ−Trr=(p1−p2)c0r−k{exp⁡(c0a−k/k)−exp⁡(c0b−k/k)}×exp⁡(c0r−kk).

It has been observed from (16) that |*T*_*rr*_ − *T*_*θθ*_| is maximum at *r* = (*e* ^2^ *b* ^*k*^)^1/*k*^ = *r*_1_; that is, yielding starts at *r* = *r*_1_; therefore,

(17) $$\begin{array}{c} {\left|T_{\theta \theta }-T_{rr}\right|}_{r=r_{1}}=\left|\frac{\left(p_{1}-p_{2}\right)c_{0}r_{1}^{-k}\exp\left(c_{0}{r_{1}}^{-k}/k\right)}{\left\{\exp\left(c_{0}a^{-k}/k\right)-\exp\left(c_{0}b^{-k}/k\right)\right\}}\right|\equiv Y. \end{array}$$

Thus pressure required for initial yielding is given by

(18) |Pi|=|e[exp⁡(−e2R0−k)−exp⁡(−e2)]k|;Pi=p1Y−p2Y=Pi1−Pi2.

For full plasticity (*C*_0_ → 0), (16) becomes

(19) $$\begin{array}{c} {\left|T_{\theta \theta }-T_{rr}\right|}_{r=b}=\left|\frac{\left(p_{1}-p_{2}\right)kb^{-k}}{\left(a^{-k}-b^{-k}\right)}\right|=Y_{1}\\ P_{f}=\left|\frac{b^{k}\left(a^{-k}-b^{-k}\right)}{k}\right|. \end{array}$$

Now, we introduced the following nondimensional quantities as

(20) R=(rb);  R0=(ab);  σrr=[TrrY];σθθ=[TθθY];  σzz=[TzzY].

The necessary pressure required for initial yielding in nondimensional form is given by

(21) $$\begin{array}{c} \left|P_{i}\right|=\left|\frac{e\left[\exp\left(-e^{2}R_{0}^{-k}\right)-\exp\left(-e^{2}\right)\right]}{k}\right|. \end{array}$$

The transitional stresses are obtained as

(22) σrr=[−Pi[exp⁡{(c0b−k/k)(R0−k−1)}−1]{exp⁡[(c0b−k/k)(R0−k−1)]−1}]−Pi2,σθθ=[−Pi{exp⁡[(c0b−k/k)(R0−k−1)]−1}] ×[(1−c0b−kR−k)exp⁡{c0b−kk(R0−k−1)}−1]−Pi2,σzz=(1−c0b−kR−k2−c0b−kR−k)(σrr+σθθ) +λc0b−kR−k(3−2c0(bR)−k)(1−c0(bR)−k)(2−c0(bR)−k)ezz,

where

(23) ezz=([−(Pi1−Pi2)2π]−∫R01b2R(1−c0(bR)−k2−c0(bR)−k)×(σrr+σθθ)dR) ×(λ∫R01c0b−k−1R−k(3−2c0(bR)−k)(1−c0(bR)−k)(2−c0(bR)−k)dR)−1.

Also, pressure required for fully plastic state is given by

(24) $$\begin{array}{c} \left|P_{f}\right|=\left|\frac{R_{0}^{-k}-1}{k}\right|;\;\;P_{f}=\frac{p_{1}}{Y_{1}}-\frac{p_{2}}{Y_{1}}=P_{f1}-P_{f2}, \end{array}$$

and fully plastic stresses are obtained as

(25) σrr=(−Pf)(R−k−1R0−k−1)−Pf2;σθθ=σr−kR−k(−Pf)(R0−k−1);σzz=kλR−k[−Pf/2π−(1/2)∫R01Rb2(σrr+σθθ)dR](R0−k−1).

*Particular Case: Nonhomogeneous Cylinder under Internal Pressure Only. *The stresses in fully plastic state are

(26) σrr=(Pf2)(R−k−1R0−k−1)−Pf2;σθθ=σrr−kR−k(Pf2)(R0−k−1);σzz=kλR−k[Pf2/2π−(1/2)∫R01Rb2(σrr+σθθ)dR](R0−k−1).

These equations in nondimensional form are the same as those obtained by Sharma [12].

## 6. Numerical Discussion

**Figure 1:**
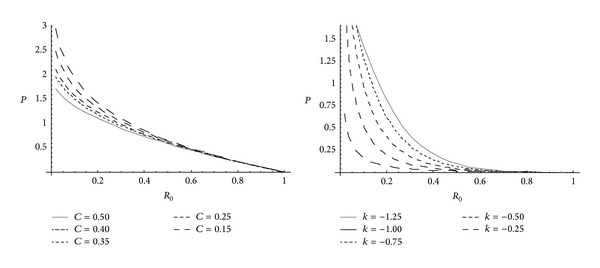
Effective pressure required for initial yielding for homogeneous and nonhomogeneous circular cylinder for different compressibility parameters.

**Figure 2:**
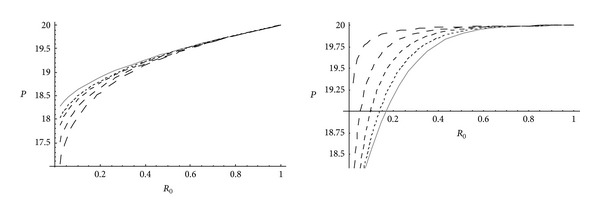
External or internal pressure required for initial yielding for homogeneous and nonhomogeneous circular cylinder (internal or external = 20) for different compressibility parameters.

To observe the combined effect of pressure on a cylinder made of homogeneous and nonhomogeneous material, graphs have been drawn between pressure and radii ratios *R*_0_ = 0.1 (0.1) 0.5. For a homogeneous (*k* = 0) circular cylinder, yielding starts at internal surface whereas for a circular cylinder made of nonhomogeneous material (*k* < 0, non-homogeneity increases radially), yielding takes place at any radius *r* where *a* < *r* < *b* depending upon values of *C*_0_ and *k*. Effective pressure is maximum at internal surface for cylinder made of nonhomogeneous as well as homogeneous material. It is seen from Figure 1 that for homogeneous cylinder, high effective pressure is required for initial yielding than that of nonhomogeneous cylinder. Also, for cylinder made of homogeneous material, effective pressure required for initial yielding is less for highly compressible circular cylinder whereas for circular cylinder made of nonhomogeneous materials, high effective pressure is required for highly compressible cylinder. It is also seen from Figure 2 that pressure (internal/external) is maximum at external surface for cylinder made of nonhomogeneous as well as homogeneous material. It is also seen that homogeneous cylinder requires high pressure for initial yielding than that of nonhomogeneous cylinder. Also, high pressure is required for initial yielding for highly compressible homogeneous cylinder whereas less pressure is required for highly compressible nonhomogeneous cylinder. 

**Figure 3:**
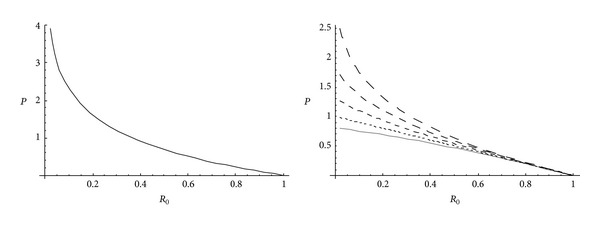
Effective pressure required for fully plastic state for homogeneous and nonhomogeneous circular cylinder for different compressibility parameters.

**Figure 4:**
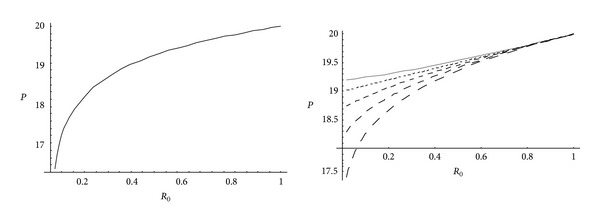
External or internal pressure required for fully plastic state for homogeneous and nonhomogeneous circular cylinder (internal or external = 20) for different compressibility parameters.

It has also been observed from Figure 3 that, for homogeneous and nonhomogeneous circular cylinder, effective pressure required for fully plastic state is maximum at the internal surface and for nonhomogeneous material less effective pressure is required for fully plastic state for circular cylinder made of highly compressible material. It is also observed that effective pressure required for fully plastic state is more for cylinder made of homogeneous material than that of nonhomogeneous material. For homogeneous/nonhomogeneous circular cylinder, pressure (internal/external) required for fully plastic state is maximum at the external surface. It has been seen from Figure 4 that for nonhomogeneous cylinder made of highly compressible material, high pressure is required for fully plastic state. It is also observed from Figure 4 that pressure required for fully plastic state is more for cylinder made of nonhomogeneous material than that of homogeneous material. 

**Figure 5:**
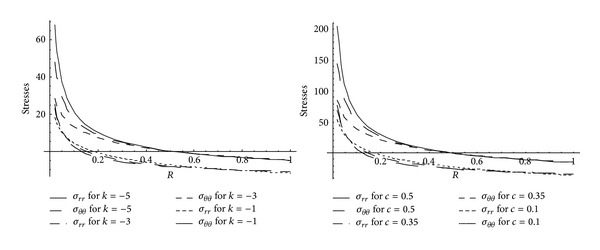
Homogeneous transitional stresses for a thick-walled circular cylinder under external pressure (*P*_2_ = 5 and 15).

**Figure 6:**
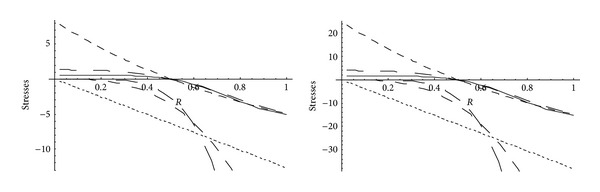
Nonhomogeneous transitional stresses for a thick-walled circular cylinder under external pressure (*P*_2_ = 5 and 15).

**Figure 7:**
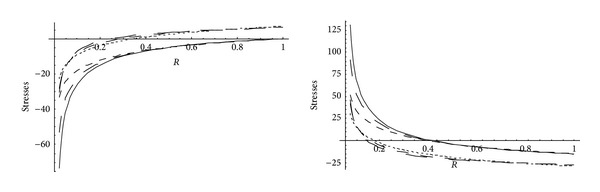
Homogeneous transitional stresses for a thick-walled circular cylinder under internal (*P*_1_ = 5) and external pressure (*P*_2_ = 0 and 15).

**Figure 8:**
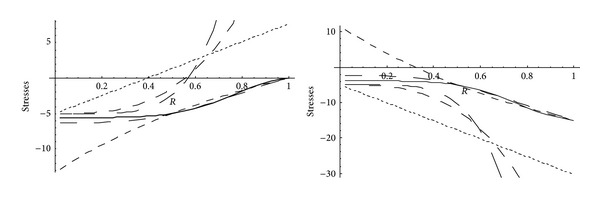
Nonhomogeneous transitional stresses for a thick-walled circular cylinder under internal (*P*_1_ = 5) and external pressure (*P*_2_ = 0 and 15).

**Figure 9:**
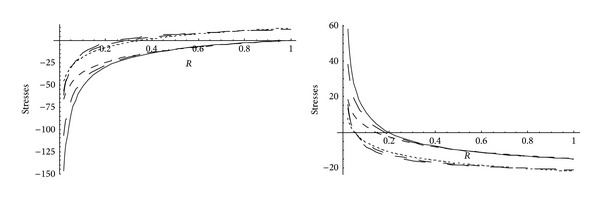
Homogeneous transitional stresses for a thick-walled circular cylinder under internal (*P*_1_ = 10) and external pressure (*P*_2_ = 0 and 15).

**Figure 10:**
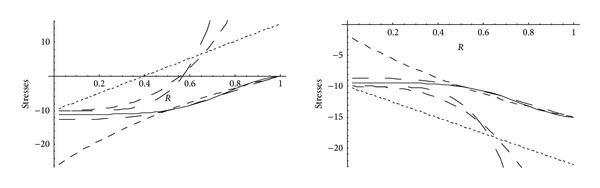
Nonhomogeneous transitional stresses for a thick-walled circular cylinder under internal (*P*_1_ = 10) and external pressure (*P*_2_ = 0 and 15).

**Figure 11:**
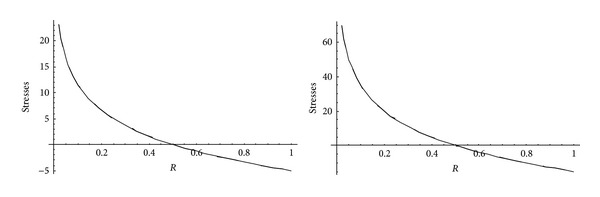
Homogeneous fully plastic stresses for a thick-walled circular cylinder under external pressure (*P*_2_ = 5 and 15).

**Figure 12:**
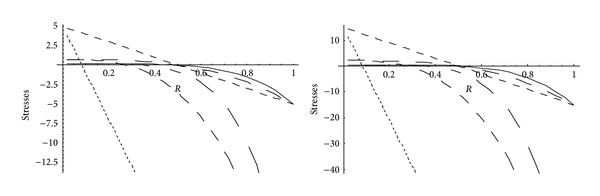
Nonhomogeneous fully plastic stresses for a thick-walled circular cylinder under external pressure (*P*_2_ = 5 and 15).

**Figure 13:**
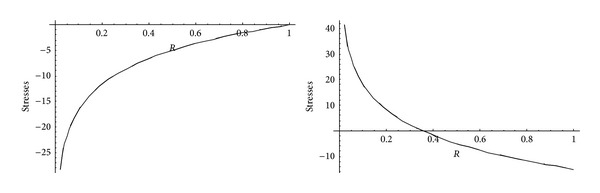
Homogeneous fully plastic stresses for a thick-walled circular cylinder under internal (*P*_1_ = 5) and external pressure (*P*_2_ = 0 and 15).

**Figure 14:**
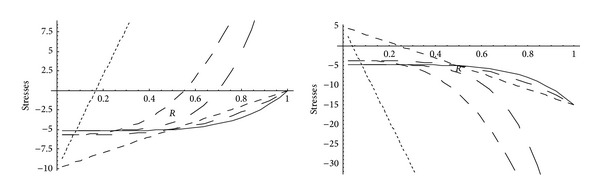
Nonhomogeneous fully plastic stresses for a thick-walled circular cylinder under internal (*P*_1_ = 5) and external pressure (*P*_2_ = 0 and 15).

**Figure 15:**
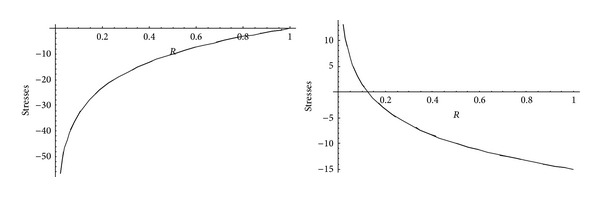
Homogeneous fully plastic stresses for a thick-walled circular cylinder under internal (*P*_1_ = 10) and external pressure (*P*_2_ = 0 and 15).

**Figure 16:**
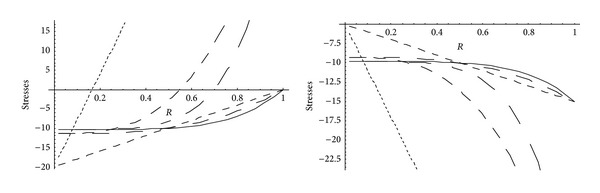
Nonhomogeneous fully plastic stresses for a thick-walled circular cylinder under internal (*P*_1_ = 10) and external pressure (*P*_2_ = 0 and 15).

From Figures 5 and 6, it has been observed that for homogeneous cylinder under external pressure only, circumferential stresses are maximum at internal surface while for nonhomogeneous cylinder, stresses are maximum at external surface. These stresses increase significantly with the increase in external pressure. With internal pressure only, as seen from Figures 7 and 8, circumferential stresses are maximum at internal surface for homogeneous cylinder while maximum at external surface for nonhomogeneous cylinder. Also it has been observed that the compressible circumferential stresses change to tensile stresses. It has also been observed from Figures 9 and 10 that with the increase in pressure, circumferential stresses increases significantly. With the increase in external pressure (greater than that of internal pressure), circumferential stresses increases. It has been observed from Figures 11 and 12 (without internal pressure) that fully plastic stresses are maximum at external surface for cylinder made of nonhomogeneous material and at internal surface for cylinder made of homogeneous material. Also, it has been observed that highly compressible cylinder is having less stress whereas less compressible cylinder is having high stress. With the increase in external pressure, stresses increases significantly. From Figures 13 and 14, it has also been observed that with the introduction of internal pressure (without external), compressible circumferential stresses are maximum at internal surface for homogeneous while at external surface for nonhomogeneous cylinder. As external pressure increase and becomes more than that of internal one, then stresses again increase but are of compressive nature. Also it has been observed that stresses increases significantly with the increase in internal pressure as can be seen from Figures 15 and 16.

## 7. Conclusions

From the above analysis we can conclude that nonhomogeneous cylinder with internal and external pressure is on the safer side of the design as compared to homogeneous cylinder because nonhomogeneous cylinder requires high pressure for initial yielding as compared to homogeneous cylinder. It has also been concluded that highly compressible nonhomogeneous cylinder is on the safer side of the design as compared to less compressible nonhomogeneous circular cylinder because highly compressible cylinder required high pressure for initial yielding as compared to less compressible nonhomogeneous cylinder, which leads to the idea of “stress saving” that minimizes the possibility of fracture of cylinder.
